# The Role of Subjective Expectations for Exhaustion and Recovery: The Sample Case of Work and Leisure

**DOI:** 10.1177/17456916221134529

**Published:** 2022-12-05

**Authors:** Victoria Schüttengruber, Alexandra M. Freund

**Affiliations:** 1 Department of Psychology, University of Zurich, Switzerland; 2 Swiss National Centre of Competence in Research LIVES – Overcoming vulnerability: Life course perspectives, Geneva, Switzerland; 3 University Research Priority Program Dynamics of Healthy Aging, University of Zurich, Switzerland

**Keywords:** exhaustion, recovery, subjective expectations, work, leisure

## Abstract

We propose a new model of exhaustion and recovery that posits that people evaluate an activity as exhausting or recovering on the basis of the subjective expectation about how exhausting or recovering activities related to a certain life domain are. To exemplify the model, we focus as a first step on the widely shared expectations that work is exhausting and leisure is recovering. We assume that the association of an activity related to a life domain associated with exhaustion (e.g., work) leads people to monitor their experiences and selectively attend to signs of exhaustion; in contrast, while pursuing an activity related to a life domain associated with recovery (e.g., leisure), people preferentially process signs of recovery. We further posit that the preferential processing of signs of exhaustion (vs. recovery) leads to experiencing more exhaustion when pursuing activities expected to be exhausting (e.g., work activities) and more recovery when pursuing activities expected to be recovering (e.g., leisure activities). This motivational process model of exhaustion and recovery provides new testable hypotheses that differ from predictions derived from limited-resource models.

Imagine Kim, a music teacher, who during his leisure time plays a song on the guitar for his friends, experiencing the activity as *recovering*. The next day, Kim is at school and plays the same song during a music lesson to exemplify this musical genre to his students. This time, Kim experiences playing the song no longer as recovering but as *exhausting*. Kim is not an exception in evaluating the same activity as more exhausting or more recovering depending on the context. In other words, neither person characteristics nor the demandingness of the activity itself appear to be the main factors determining how exhausting or recovering it is experienced. What, then, affects whether an activity is experienced as exhausting or recovering? To address this question, we propose a new model of exhaustion and recovery that builds on the assumption that people expect activities to exhaust them when pursued in certain contexts (but not in others) and that this expectation leads to preferential information processing of indicators of exhaustion (or recovery) by guiding attention toward signs of exhaustion (or recovery) and the accumulation of exhaustion-related (or recovery-related) evidence that then leads to the perception of being exhausted (or recovered). We center on the sample case of the widely shared expectations about exhaustion and recovery of work- and leisure-related activities to present and exemplify the new model.

Our central hypothesis is that people hold subjective expectations about how exhausting or recovering activities related to certain life domains are. Arguably one of the most widely shared expectations related to exhaustion and recovery is that work-related activities are exhausting and leisure-related activities are recovering. This expectation holds as a general tendency, although some work-related activities might not be particularly effortful (e.g., searching the Internet for pictures illustrating a presentation), and some leisure activities are quite demanding (e.g., playing chess against a formidable opponent, running a marathon). Interestingly, some evidence suggests that people expect—and, as a consequence, also experience—the same activity to be more exhausting and less recovering when it is framed as work compared with leisure (Freund, 2018). This article presents a process model that integrates the potential effects of subjective expectations with the experience of an activity as exhausting or recovering. We argue that the expectations of work-related activities as exhausting and leisure-related activities as recovering selectively guide the attention toward signs of exhaustion or recovery, respectively (Nickerson, 1998; Zion & Crum, 2018). In other words, when pursuing a work-related activity, people expect to become exhausted, guiding their attention to indicators of exhaustion, which, in turn, likely leads to an accumulation of evidence in favor of the expectation (i.e., of signs of exhaustion). The opposite pattern should occur when engaging in a leisure-related activity: On the basis of the subjective expectation that leisure-related activities are recovering, people are assumed to pay particular attention to indicators of recovery, leading to an accumulation of evidence in favor of the expectation to experience indicators of recovery.

What are the indicators of exhaustion and recovery? Cardini and Freund (2019) argued that perceived opportunity costs, mood, and time perception determine the degree of perceived exhaustion or recovery. Subsequent research has provided some empirical evidence for these indicators of exhaustion and recovery (Cardini & Freund, 2020, 2021).

Before diving deeper into this model, we start this article by addressing how people categorize a given activity as belonging to a certain life domain. Focusing on work and leisure, we first ask which cues people use to categorize an activity as belonging more to work or to leisure. Then, we turn to the question of how this categorization impacts the experience of exhaustion and recovery. The first question concerns the antecedents and the second the consequences of perceiving an activity as belonging to work or leisure. The aim of the resulting model is to provide insights into the role of subjective expectations on the experiences of exhaustion and recovery. This model not only allows testable hypotheses to be derived but also contributes to the research area of boundary management and more broadly to the fields of motivation, occupational psychology, and health psychology.

## Managing Multiple Tasks in Work and Leisure

Although people of all ages pursue multiple goals with limited resources such as time or money, the phases of young and middle-aged adulthood are particularly challenging regarding the management of multiple tasks and demands in different life domains (e.g., career goals, childcare, caring for parents, hobbies; Freund, 2020). In their everyday lives, people strive for a successful work–nonwork balance to deal with exhaustion and recovery (Bennett et al., 2018; Knecht et al., 2016). Work–nonwork balance is an abstract and vaguely defined notion of an optimal ratio between (paid) work and nonwork (Guest, 2002). People segment their everyday lives into life domains (Edwards & Rothbard, 2000) and categorize activities as belonging to one or more life domains (Knecht & Freund, 2016). Work mostly describes paid activities that people pursue in exchange for monetary compensation. To maintain this exchange, one needs to fulfill certain obligations. Unpaid work is also characterized by obligations but does not imply monetary rewards. Examples are many work-related activities (e.g., unpaid overtime, commutes, volunteering), household chores, or childcare (e.g., Sonnentag, 2001; Voss, 1967). Although household chores are for most people unpaid, very few would count them as a leisure activity. Leisure refers to activities that are free from obligations (i.e., discretionary time). Not surprisingly, then, perceptions of high autonomy are often related to whether an activity is perceived as leisure (Knecht et al., 2016). The aspect of autonomy might contribute to interindividual differences in what is considered a leisure activity (Newman et al., 2014): Whereas some people might feel that singing in a choir is a leisure activity, others might perceive it more as a work-like activity because of the high degree of commitment to show up for practice and performances.

Which cues do people use to categorize an activity as belonging more to work or leisure? Despite the abundant research on work–nonwork balance, only little is known about the cues that people use to distinguish between work activities and leisure activities (Inzlicht et al., 2014). According to research on boundary management (Allen et al., 2014), people draw physical, temporal, and psychological boundaries around life domains on a continuum from separation to integration (i.e., complete overlap). Separation consists of clear boundaries that maximize the difference between life domains. Blurred boundaries minimize this difference and create integration (Ashforth et al., 2000). It remains poorly understood which cues people use to segment the stream of ongoing activities into units, which they categorize as belonging to one or several different life domains.

Residual (or antithetic) definitions of leisure define leisure as the opposite of work (i.e., the residual time that is not dedicated to work; Voss, 1967), ignoring the potential overlap between the two life domains. From an economic perspective, work entails monetary rewards, and leisure implies a forgone income (Kool & Botvinick, 2018), although skills acquired during leisure (e.g., learning a new language) may translate at some point into monetary compensation, and not all work is paid (e.g., revising a manuscript on the weekend). Experiential definitions of leisure (e.g., Iso-Ahola, 1979; Newman et al., 2014) identify autonomy, freedom from work-related obligations, enjoyment, social connection, and activities done solely for their own sake as being characteristic of leisure.

However, these characteristics may also describe many work activities. For instance, many people enjoy their work, feel a social connection to their coworkers, and experience high autonomy at work. In fact, most experiential characteristics of activities are not exclusive to either work or leisure. For example, work-related activities may or may not require the investment of effort, and people pursue many leisure activities (e.g., running a marathon) with sustained investment of high effort (Stebbins, 2017). The same applies to how much fun or how boring an activity is (Csikszentmihalyi & LeFevre, 1989; Iso-Ahola, 1979): Leisure is not always fun and interesting, and work is not always aversive and boring. For example, learning a new programming language for a work-related project or training for a marathon as a leisure activity may be pleasant one time but exhausting another. Moreover, people primarily look for work they consider to be interesting, and they tend to quit their jobs when work is not fun over longer periods of time (Hoff et al., 2020). Thus, equating leisure with effortless fun activities and work with effortful and mostly aversive activities does not adequately capture their categorization. Which cues, then, do people use to categorize an activity as belonging more to work or leisure? We return to this question after reviewing the literature on concepts of exhaustion and recovery.

## Conceptual Clarifications of Exhaustion and Recovery

Reviewing the literature on the concepts of exhaustion and recovery leads to the field of work–nonwork balance (or, as it is often called, work–life balance) and more generally to the research area of motivation. The first research line mirrors associations of work with exhaustion and leisure with recovery (e.g., Sonnentag et al., 2017), and the second one informs our process model of exhaustion and recovery (Cardini & Freund, 2019; Kurzban, 2016).

How can we define exhaustion and recovery? Exhaustion denotes “an aversion to continue with the present activity and a decrease in the level of commitment to the task at hand” (Boksem & Tops, 2008, p. 126) and occurs after or during prolonged activity performance. However, exhaustion does not necessarily lead to performance decrease, for example, when people invest additional (compensatory) effort (e.g., van der Linden, 2011). Recovery is the counterpart: It reduces or removes signs of exhaustion (Cardini & Freund, 2019).

The proposed process model of exhaustion and recovery addresses temporal signs of exhaustion, as opposed to chronic or clinically relevant experiences of exhaustion (e.g., such as in burnout or depressive episodes) that result from accumulated processes of exhaustion (e.g., Demerouti et al., 2001, 2021; Freudenberger, 1974; Maslach et al., 2001). For instance, the job demands-resources model (e.g., Demerouti et al., 2001) suggests that chronic states of work-related exhaustion stem from stable conditions such as high job demands over long periods of time. Whether our model could also be applied to long-term exhaustion (and recovery) is an interesting question; however, it is beyond the scope of this article.

### Recovery from work-related exhaustion

The literature on work–nonwork balance is primarily concerned with how people can recover from work-related exhaustion during nonwork time (Bennett et al., 2018; Sonnentag et al., 2017). Recovery can be defined as “the process of reducing or eliminating physical and psychological strain symptoms that have been caused by job demands and stressful events at work” (Sonnentag & Fritz, 2015, p. 72). Work-related exhaustion^1^ denotes “extreme tiredness and reduced functional capacity that is experienced during and at the end of the workday” (Frone & Tidwell, 2015, p. 274). The absence of work-related factors that have caused exhaustion is seen as a prerequisite for recovery (Sonnentag, 2012). According to this perspective, leisure provides the greatest potential to achieve recovery from work-related exhaustion (Kuykendall et al., 2015; Newman et al., 2014). Thus, this literature implies a strong association between work and exhaustion on the one hand and leisure and recovery on the other.

Frameworks of recovery mirror associations of work with exhaustion and leisure with recovery. According to the effort-recovery model (Meijman & Mulder, 1998), work demands cause acute load reactions, including physiological (e.g., cardiovascular activation) and psychological (e.g., exhaustion, performance decrease) responses to effort exertion at work. When people refrain from work-related demands, recovery reverses these acute load reactions. According to Sonnentag (2012), psychological detachment, defined as the experience of “switching off” (i.e., disengaging) from work-related activities, thoughts, and locations during nonwork time, is central for recovery from work. Supporting this hypothesis, studies show that detachment attenuates and removes exhaustion from work that otherwise would carry over to nonwork time, impair recovery, and harm physical and psychological health in the long run. Thus, this literature implies that people should separate work and leisure to recover adequately from work-related exhaustion (e.g., Sonnentag, 2012, 2018).

Taken together, frameworks of work-nonwork balance and recovery imply the conceptual distinction of *exhausting work* and *recovering leisure.* These expectations are likely also reflected in people’s subjective expectations: When engaging in an exhausting leisure activity, people might describe it as work-like (on the basis of their expectation that work, but not leisure, is exhausting), and when a work-related activity was recovering, they might say that “this was no work at all” (on the basis of their expectation that leisure, but not work, is recovering). The categorization of activities as belonging more to work or leisure depends on the relative importance of the cues (e.g., the work goal might outweigh the impact of high autonomy or vice versa). Interindividual differences in the relative importance of the cues explain why a given activity is more like work for some people and more like leisure for others. Highlighting the impact of this subjective categorization, we posit that people expect—and experience—the same activity as more exhausting and less recovering when they categorize the activity as work compared with leisure (Freund, 2018).

### Motivational accounts of exhaustion and recovery

Arguably the most prominent approaches to exhaustion and recovery are resource models that posit that exhaustion is the result of a depletion of energy, be it physical, cognitive, or motivational energy (e.g., Baumeister et al., 1998). Assuming that exhaustion indicates the depletion of resources, its main function would be to conserve energy to protect the organism against states that can no longer guarantee basic functioning. This seems highly plausible. However, resource models have been criticized both on theoretical and empirical grounds (e.g., Carter et al., 2015; Inzlicht et al., 2014; Navon, 1984). Theoretically, it is unclear to which resource these models refer; beyond physical energy (i.e., how many calories walking, running, or swimming a certain distance burn), the concept of energy remains mostly undefined and fuzzy (Cardini & Freund, 2021). Reviewing the empirical literature, D. R. Evans et al. (2016) concluded that even after extremely high investment of physical energy, people are typically not anywhere near levels of depletion threatening brain functions and survival. Regarding motivation, after typical depletion procedures, people are able to continue engaging in a task when incentivized (e.g., Inzlicht et al., 2014). Moreover, people who are led to believe that motivational resources are unlimited and do not deplete with use show fewer signs of exhaustion compared with people who are led to believe that resources are limited and deplete with use (Job et al., 2015).

What, then, could be the function of exhaustion? One possibility is that exhaustion serves as a signal expressing that the current activity yields more costs than benefits. When costs exceed benefits, it is adaptive to disengage from it and switch to another activity. According to Cardini and Freund (2019), exhaustion is a signal to disengage from an activity that does not (or no longer) result in the desired outcomes. When people were asked what exhaustion feels like for them, many listed that they can no longer concentrate on the activity at hand and experience negative mood and a lengthening of time (Cardini et al., 2018; Cardini & Freund, 2019, 2021). These expressions of exhaustion can be conceptualized as indicators of an unfavorable cost-benefit ratio. Problems in concentrating on a given task and instead thinking about other activities represent opportunity costs (i.e., forgoing an alternative to the current activity; Kurzban et al., 2013). In addition to opportunity costs, a worsening mood indicates that the current activity does not yield the targeted outcomes, or does so more slowly than expected (Carver & Scheier, 2002). According to the mood-as-information model, negative mood is a stop signal that leads people to scrutinize the current situation and activity for things that are not going well (Clore & Huntsinger, 2007). When continuing to engage in an activity becomes aversive, people are likely to prioritize more rewarding activities (Boksem & Tops, 2008; Inzlicht et al., 2014; Kurzban et al., 2013). Similarly, time perception is sensitive to the cost–benefit ratio of engaging in an activity: Time seems to fly when making progress and to slow down when being stuck (Zakay, 2014). In the extreme, people experience flow when being engrossed in an activity that is simultaneously demanding and for which people have high competence (Nakamura & Csikszentmihalyi, 2009).

Cardini and Freund (2019) conceptualized recovery in the same way: When engaging in an activity to recover, a worsening of mood, experiencing opportunity costs (i.e., starting to think about alternative activities), and a lengthening of experiencing time serve as indicators of recovery. These experiences motivate people to switch to a different activity. In other words: When people are sufficiently recovered or an activity no longer contributes to recovery, the cost–benefit ratio turns unfavorable, which prompts disengagement from the activity. This model is empirically supported in a series of studies by Cardini and Freund (2020, 2021). Building on the model by Cardini and Freund (2019), we further elaborate here on the role of subjective expectations for exhaustion and recovery.

## Effects of Subjective Expectations

The term “leisure” is almost synonymous with relaxation, and the term “work” is almost synonymous with effort and exhaustion, reflecting the subjective beliefs that work activities are exhausting and leisure activities contribute to recovery. Moreover, the popular press on work–nonwork balance predominantly recommends separating work and leisure to achieve sufficient recovery from work-related exhaustion. These recommendations skyrocketed during the COVID-19-induced lockdowns in many countries that necessitated working from home (for Germany, see Vetter, 2020; for Switzerland, see Siegenthaler, 2020; for Japan, see Bloomberg, 2020; for India, see Chakravarty, 2020; for the United Kingdom, see Lawrie & Parry, 2020; Taylor, 2020; for the United States, see Loggins, 2020; Miller, 2020). Such recommendations likely reinforce expectations of work as exhausting and leisure relaxing. What are the consequences of such expectations? In the following, we outline several research lines that investigate the impact of subjective expectations on people’s psychological functioning and apply them to the field of exhaustion and recovery.

An expectation^2^ is a “belief about the future based on a prediction of what is most likely to happen” (Zion & Crum, 2018, p. 5) and of “the nature and likelihood of future states” (p. 10). Beliefs influence people’s experiences, emotions, behaviors, motivation, and physiological processes (Zion & Crum, 2018). Most prominently, placebos (i.e., substances or procedures that do not contain active pharmacological ingredients or surgical procedures) can elicit the expected medical treatment effects (Stewart-Williams & Podd, 2004). Psychological research has investigated the effects of expectations in different areas such as implicit theories about the nature of human characteristics as fixed or malleable (e.g., Dweck, 1999; Zedelius et al., 2017) or health-related mindsets (e.g., Crum & Zuckerman, 2017; Crum et al., 2013). For example, hotel room attendants framing their typical cleaning activities as “good exercise” showed objective health-related benefits (weight loss, cardiovascular functioning), but not attendants who engaged in the very same activities but did not receive the experimental instruction (Crum & Langer, 2007). Research on perceived depletion (Clarkson et al., 2010) and implicit theories about willpower (Job et al., 2010) illustrates that people’s performance decreases only if people subjectively expect to have a limited amount of willpower available that is depleted by exerting effort.

Building on this evidence, we propose that framing an activity as either work or leisure activates the expectations of exhaustion or recovery, respectively. In a similar vein, Eden (2001) discussed the impact of expectations of recovery that function as self-fulfilling prophecies during time off work. Moreover, Tonietto et al. (2021) showed empirically that people enjoyed their leisure activities less and reported less favorable mental health when they believed that leisure was wasteful and unproductive.

Two studies provide preliminary evidence supporting the subjective expectations of exhausting work and recovering leisure (Study 1: *N* = 49; Study 2: *N* = 315; Freund, 2018). Participants rated the degree of exhaustion and recovery in four activities when performed during work or leisure time (meeting with others, physical exercise, reading, surfing the Internet). Both studies showed a pronounced difference in the self-reported exhaustion or recovery associated with the activities at work versus during leisure: Participants perceived the same activity as being more exhausting when associated with work and as more relaxing when associated with leisure. These findings raise the question of how the categorization of an activity as work results in feeling more exhausted and the categorization of the same activity as leisure in feeling more recovered. We address this question in the next section.

## Exhausting Work and Recovering Leisure: a Process Model

We hypothesize that people expect—and experience—the same activity to be more exhausting and less recovering when it is framed as work compared with leisure (Freund, 2018). Alternatively, people might generally invest more effort when performing an activity during work than during leisure and be more relaxed when doing something during leisure than during work. However, everyday life yields many examples of effortful and relaxing activities in both life domains (Sonnentag, 2001). In the process model we propose here, we conceptualize the antecedents of the categorization of activities as belonging to the life domains of work or leisure and its consequences on the experience of exhaustion and recovery. Several testable hypotheses can be derived from the model, setting the stage for future research.

In line with expectancy effects (e.g., Zion & Crum, 2018), we assume that people hold expectations (e.g., about how exhausting or recovering activities in certain life domains are) that selectively guide attention toward specific experiences (e.g., perceiving signs of exhaustion) while neglecting others (e.g., neglecting signs of recovery). Note that we propose that people do not hold expectancies about how exhausting or recovering every singly activity is per se (e.g., preparing a book review) but that the expectation and experience of exhaustion and recovery primarily depends on whether an activity (e.g., reading) is perceived as belonging to work (e.g., for teaching) or to leisure (e.g., for a book club presentation).

To explain why the same activity might be categorized as work or leisure, we propose several cues for the categorization of activities. Importantly, we posit that the categorization is causal for experiencing exhaustion or recovery and hold that people do not infer that an activity belongs to work or leisure on the basis of their assessment of how exhausting or recovering it is. In other words, we claim that the categorization (rather than the activity itself) causally impacts whether the activity is more exhausting or recovering and selectively directs attention toward motivational indicators of exhaustion (in work-related activities) or recovery (in leisure-related activities).

### Antecedents of the segmentation between work and leisure

Before addressing the consequences of subjective expectations for the experience of exhaustion and recovery, we target a more basic question: On what basis do people categorize an activity as belonging more to work or to leisure? We propose a set of cues that people might use for this purpose. These cues represent central qualities to distinguish between work and leisure activities in general and thereby not only apply to specific types of activities.

According to research on event perception, people segment the flow of continuous experience into events (Zacks, 2020). They monitor several cues (e.g., sensory cues, goals) to segment their ongoing experience into different activities (as events). Applied to the categorization of activities as belonging to work or leisure, we propose several cues that people use to segment the stream of ongoing activities into work or leisure units: the goal of the activity (e.g., “I have a beer with my colleague to strengthen my professional network” vs. “I have a beer with my colleague to have fun”), monetary compensation (i.e., getting paid for the activity or not), perceived autonomy (e.g., “I have to learn the piano piece to perform at the concert” vs. “I want to learn the piano piece by the end of the week”), location (e.g., at the office vs. at home), time (e.g., during the typical work day vs. on the weekend), and social partners (e.g., colleague vs. friend). In what follows, we present and discuss each cue separately. We acknowledge that an extensive list would also include more idiosyncratic cues such as clothing (e.g., business suit vs. jogging pants), equipment (e.g., office desktop computer vs. private laptop), and maybe even auditory or olfactory cues (e.g., school bell ending a teaching session vs. sound of one’s child’s video games; odor of chemicals vs. personal diffuser blend). The outlined cues derive from the literature and empirical research on leisure (e.g., Newman et al., 2014), boundary management (Ashforth et al., 2000), and multiple goal pursuit (e.g., Freund & Knecht, 2016).

*Goals* are “cognitive representations of personally desired (or dreaded) states to be approached (or avoided) through action” (Freund et al., 2012, p. 281). Goals direct and regulate people’s behavior across situations, and they “structure and organize behavior over time into meaningful action units” (Freund & Riediger, 2006, p. 353). Note that the goal might not suffice to categorize an activity because a single activity might simultaneously serve different life domains (Knecht & Freund, 2016).

*Monetary compensation* (e.g., monthly wage) received in exchange for performing certain activities indicates work (Edwards & Rothbard, 2000; Heyman & Ariely, 2004; Kool & Botvinick, 2018; Voss, 1967). Again, monetary compensation might be not sufficient to categorize an activity as work or leisure because some work-related activities are unpaid (e.g., professional training, unpaid overtime, commute to work, voluntarism; Guest, 2002).

*Autonomy* refers to the degree of perceived freedom in choosing to perform an activity and how to execute it (e.g., for how long, how accurately). Autonomy also entails (the lack of) perceived freedom regarding the duration, time, and location of pursuing an activity; the number of repetitions; the required accurateness; and the performance standards and constraints. High autonomy is typical for leisure activities (Inzlicht et al., 2014; Newman et al., 2014). For example, wanting (vs. having) to read a book for 2 hours typically contributes to perceiving the activity as belonging more to leisure than work. In fact, framing the same activity as work (rather than leisure) decreases perceived autonomy (Rom et al., 2020), although many leisure activities involve commitments (e.g., team sports) and constraints (e.g., the availability of necessary equipment; Knecht et al., 2016; Stebbins, 2017).

*Location* refers to the physical location (e.g., tennis court, home, workspace) where the activity is pursued. *Time* refers to the time of the day (e.g., typical office times) or weekday when the activity is pursued. However, flexible work arrangements (e.g., working from home) and the usage of wireless technologies (e.g., phone, email) render physical and temporal demarcations less reliable for the segmentation between work and leisure (Ashforth et al., 2000; Butts et al., 2015).

*Social partner(s)* involves joint-activity pursuit with people associated with work or leisure (e.g., family, friends vs. colleagues, customers). The mere presence or absence of these people serves as a cue for the categorization of an activity. However, some people indicate work and leisure (e.g., colleagues who are also friends), and the absence of social partners does not signal whether an activity belongs to work or leisure (Edwards & Rothbard, 2000).

**Fig. 1.**
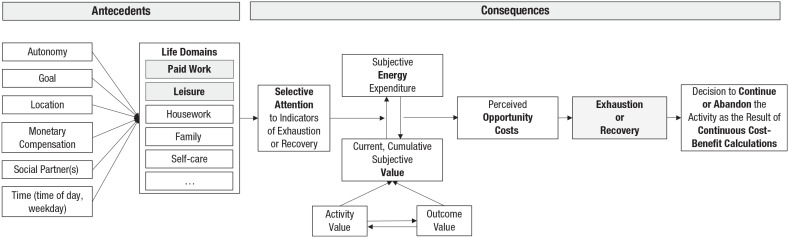
Antecedents of the categorization of an activity as work or leisure and its consequences for the experience of exhaustion and recovery. People use cues to categorize an activity as belonging more to work or to leisure. The related expectations of work being exhausting and leisure recovering are associated with selective attention to signs of exhaustion and recovery. Moreover, the subjective value together with the perceived energy expenditure are associated with the perceived opportunity costs that, in turn, are experienced as exhaustion or recovery. These experiences lead to the decision to continue with or disengage from the activity.

Taken together, these cues are proposed to function as the basis for the categorization of an activity as belonging to work or leisure (see Fig. 1). The categorization of an activity likely depends on several cues. The relative importance of each cue may depend on individual circumstances. For instance, for a person who works exclusively in the office and never engages in leisure-related activities while being in the office, the location might be sufficient for the categorization. In contrast, a person who often works on their couch at home and researches vacation spots while in the office, the location might be less indicative. Evidence from comprehensive experiments systematically varying the cues revealed that the goal is the most impactful cue for the categorization of activities as work- or leisure-related (Schüttengruber & Freund, 2022). Idiosyncratic effects in the weight that people ascribe to each cue underscore that there are no “objective” work or leisure activities. In short, we propose that the prototypical work (leisure) activity is paid, serves a work (leisure) goal, is associated with low (high) autonomy, engagement during (outside) their “usual” workhours at (outside) their “usual” (depending on the profession) workplace, with social partners associated with work (leisure).

What happens if not all cues consistently point toward work or leisure? Varying the cues (e.g., social partner) changes to what degree an activity (e.g., making coffee) is perceived more like work (e.g., for a customer) or leisure (e.g., for a friend). Work and leisure are not opposites of one dimension but rather represent two separate dimensions. Activities can belong to work and leisure to varying degrees, and the relative importance of the cues informs the categorization of the activity as being more work-related or more leisure-related. Accounting for interindividual differences in the cue importance, the impact of one cue (e.g., low autonomy) might outweigh the impact of the others (e.g., at home, on a Sunday, the goal to have a nice garden) for some people but not for others. Consequently, people can differ in their subjective categorization of the same activity, which impacts the expectation and experience of the same activity as being more exhausting (when categorized as work) or more recovering (when categorized as leisure).

### Consequences of the categorization of an activity as work or leisure

Building on the potential effects of the hypothesized expectations that work is exhausting and leisure is recovering, the second part of the process model addresses the consequences of the categorization of an activity as belonging to work or leisure. For this purpose, we outline dynamic relationships between several motivational factors: selective attention to signs of exhaustion or recovery, the subjective value of the activity itself and the anticipated outcome, perceived amount of energy expenditure, and the opportunity costs associated with being engaged in this—compared with another potential—activity. Figure 1 presents the process model distinguishing between the antecedents of the categorization of an activity as work or leisure and its consequences for exhaustion and recovery. Table 1 lists the hypotheses that derive from the sample case of expectations about exhaustion and recovery in the work and leisure context.

**Table 1.** Hypotheses on Expectations about Exhaustion and Recovery in Work and Leisure

Hypothesis	Model part
An activity is categorized as belonging to work, leisure, or another life domain depending on (a) the goal it serves, (b) the expectation of monetary compensation, (c) the degree of perceived autonomy, (d) the location, and (e) the time (time of day, weekday) of activity pursuit, as well as (f) the presence or absence of social partners.	Antecedents
An activity categorized as work is more exhausting and less recovering than the same activity categorized as leisure.	Consequences
An activity categorized as leisure is less exhausting and more recovering than the same activity categorized as work.	Consequences
Opportunity costs are positively related to exhaustion and negatively related to recovery.	Consequences

Note: Hypotheses were derived from the model referring to the sample case of expectations about exhaustion and recovery in the work and leisure context.

## Selective Attention for Signs of Exhaustion and Recovery

We assume that the subjective expectations that work-related activities are associated with exhaustion and leisure-related activities with recovery lead to selective attention to signs of exhaustion or recovery. In other words, these expectations shape top-down perceptions of exhaustion and recovery (e.g., Zion & Crum, 2018). Activities categorized as work activate attention to signs of exhaustion, whereas activities categorized as leisure activate selective attention to signs of recovery.

Research on legitimacy in the labor context (Semmer et al., 2019) provides evidence for the assumption that people selectively attend to different aspects of an activity depending on the life domain. Semmer et al. (2019) showed that what people consider illegitimate depends on their profession and that stress reactions arise only if expectations of legitimacy are violated. According to this research, an activity is not stressful per se but becomes stressful if people perceive it as unnecessary and unreasonable (for the given profession). For example, Doctors Without Borders accept working conditions (e.g., low hygiene, poor equipment) when deployed in crisis regions that they would consider unacceptable in Western countries (Semmer et al., 2019). If workload violates organizational norms for time pressure, feelings of illegitimacy contribute to strain and depressive symptoms regardless of the actual workload. Norms for time pressure vary by profession (Ford & Jin, 2015). Similarly, what people consider constraining to their work performance results from their standards set as necessary to fulfill their work activities (Pindek & Spector, 2016). Depending on the categorization of patients’ recovery status, nurses hold different standards for legitimate (e.g., support the healing process) and illegitimate (e.g., behavior like hotel guests) requests (Semmer, 2000).

Similarly, we propose that people hold different standards of exhaustion and recovery in the life domains of work and leisure associated with selective attention to signs that indicate either exhaustion (for work-related activities) or recovery (for leisure-related activities). People tend to seek, process, and interpret evidence that is consistent with their expectations. In contrast to scientific hypothesis testing, people do not try to falsify their expectations by seeking counterevidence. This confirmation bias is reflected in people’s tendency to asymmetrically collect evidence in the service of confirming their expectations—in this case, that activities perceived as belonging more to work are exhausting and activities perceived as belonging more to leisure are recovering (J. S. B. T. Evans, 2016; Nickerson, 1998).

So far, we have argued that the categorization of an activity as belonging more to work or leisure activates the widely shared associations of work with exhaustion and leisure with recovery. These expectations selectively guide people’s attention to signs of either exhaustion (in work-related activities) or recovery (in leisure-related activities). We now turn to the factors that might contribute to the perceived *degree* of exhaustion and recovery.

### Subjective value and energy expenditure

We propose that the relation between the subjective value of a given activity and the subjective energy expenditure constitutes one of the factors affecting the degree of experienced exhaustion (or recovery). This relation is associated with the perceived opportunity costs of the activity. An activity carries a current, cumulative subjective value at a given moment (Berkman et al., 2017) that fluctuates over time during activity pursuit. Here, value refers to the *subjective utility* of the activity. The subjective value does not result from the categorization of an activity as work or leisure but instead integrates the intrinsic value of the activity itself (i.e., enjoyment) and the value of its outcome (i.e., the consequences).

The activity value depends on the degree to which the pursuit of activity is in itself enjoyable and gives people a sense of competence and control. People perceive activities with a high activity-oriented incentive (German: “tätigkeitszentrierter Anreiz”) as rewarding, enjoyable, and “an end in itself” (Rheinberg, 2010). The incentive is located “inside” the very pursuit of the activity (Locke & Schattke, 2019), which becomes similar to goal attainment (Kruglanski et al., 2018). Freund et al. (2010; Freund & Hennecke, 2012) found that people who focus on the process of goal pursuit are more likely to maintain engagement in difficult goals such as exercising regularly or keeping a diet and experience more positive mood. This suggests that the activity value, a concept that has been mostly overlooked in models of subjective value, plays an important role for the evaluation of an activity. The second aspect of the subjective value is the outcome value. The outcome value represents the subjective evaluation of the outcome of a given activity (i.e., end states and consequences of activity pursuit). It depends on the costs (invested effort) on the one hand and the desirability of the outcome on the other (Locke & Schattke, 2019). People pursue activities with a high outcome-oriented incentive (even if these activities are aversive) because their consequences function as incentives (German: “zweckzentrierter Anreiz”). The incentive is located “outside” the activity (Rheinberg, 2010).

Taken together, the activity value describes to what degree one enjoys the process of activity pursuit itself. The outcome value describes the value derived from the activity in relation to the end states and consequences. Activity value and outcome value are integrated into an overall (current and cumulative) subjective value of the activity that fluctuates over time and results in the opportunity costs of the current activity (Kurzban, 2016). People do not necessarily perceive opportunity costs as such, but they might be expressed as interfering thoughts about alternative activities, a decreasing motivation to continue with the current activity, a worsening of mood, and a slowing of the perceived passing of time (Cardini & Freund, 2019, 2020, 2021).

#### Subjective energy expenditure

Energy expenditure^3^ is the subjective (rather than physiological) amount of energy that one puts into the pursuit of an activity. According to motivational intensity theory (e.g., Gendolla et al., 2019; Richter et al., 2016), energy mobilization increases proportionally to the levels of subjective task difficulty, as long as people perceive success as possible and worthwhile. The importance of successful activity pursuit determines an upper limit of effort mobilization. In line with this theory, we propose that the subjective value impacts energy expenditure by setting the upper limit of energy mobilization. The subjective value serves as a reference point and determines the maximum amount of energy that people are willing to invest into a given activity. This maximum level of energy mobilization is compared to the actual amount of perceived energy expenditure (Berkman et al., 2017; Richter et al., 2016). In short, the subjective value determines energy expenditure. However, there is evidence showing that the opposite direction is also possible: Energy expenditure can increase or decrease the subjective value (for a review on the effort-value association, see Inzlicht et al., 2018; for an earlier discussion, see Lewis, 1965).

#### Ratio of value and energy expenditure

Energy expenditure and subjective value influence each other and result in a ratio of maximum and perceived amount of energy expenditure. There are three scenarios of the bidirectional relationship. In the first scenario, as outlined above, the subjective value determines the amount of energy that people are willing to invest in an activity. If the subjective value of an activity increases, people are willing to expend more energy (Richter et al., 2016). Alternatively, energy expenditure can decrease or increase the subjective value of a given activity. In the second scenario of the bidirectional relationship between energy expenditure and subjective value, energy expenditure represents *costs* (e.g., intrinsic costs, Kool & Botvinick, 2018; energetic costs, Boksem & Tops, 2008) of a—largely undefined—scarce resource (Kurzban, 2016; Shenhav et al., 2017) that decreases the subjective value. People strive to minimize the costs of energy expenditure by avoiding physical (Hull, 1943) and cognitive effort (Kool et al., 2010). They prefer activities with low (rather than high) expenditure. In cost-benefit analyses, rewards can counteract the costs of energy expenditure (Kool & Botvinick, 2018).

In a third scenario, energy expenditure enhances the activity value or the outcome value. People often invest effort in the absence of (monetary) rewards (Heyman & Ariely, 2004) and enjoy the process of effort exertion (e.g., playing chess). An increase in activity value occurs when value derives from the process of effort exertion (Inzlicht et al., 2018). Examples are the need for cognition (i.e., preference for mentally effortful activities; Cacioppo & Petty, 1982), learned industriousness (i.e., repeated rewards for effort turn effort exertion into a reward; Eisenberger, 1992), and flow (i.e., being fully immersed in an activity as an end in itself; Nakamura & Csikszentmihalyi, 2014). An increase in outcome value occurs when value derives from the outcome of effort exertion (Inzlicht et al., 2018). Examples are effort justification (i.e., retrospective justification of effort by enhancing the value of outcomes; Aronson & Mills, 1959), the effort heuristic (i.e., ascribing higher quality to products if the production process involved high effort; Kruger et al., 2004), and the so-called IKEA effect (i.e., ascribing higher value to self-assembled products compared with preassembled products; Norton et al., 2012).

The relationship between the subjective value and energy expenditure results in a favorable or unfavorable ratio of maximum and perceived amount of energy expenditure. A favorable ratio indicates that perceived energy expenditure is lower than or equal to the maximum energy expenditure. An unfavorable ratio indicates that perceived energy expenditure exceeds maximum energy expenditure. Changes in this ratio influence the degree of perceived opportunity costs. A favorable ratio results in low opportunity costs, and an unfavorable ratio results in high opportunity costs. Opportunity costs fluctuate depending on the dynamic character of the ratio of subjective value and energy expenditure.

### Opportunity costs

In line with motivational accounts of exhaustion and recovery (Cardini & Freund, 2019; Kurzban et al., 2013), we propose that perceived opportunity costs are indicators of exhaustion (or recovery) and signal to the person to disengage from the current activity and engage in an alternative activity with higher value. Opportunity costs express mostly unconscious cost–benefit computations that revolve around the continuation of the current activity compared with the value of the foregone alternative activities. Depending on whether people expect an activity to exhaust or recover them, low or indiscernible opportunity costs indicate low exhaustion (in activities categorized as work) or high recovery (in activities categorized as leisure). Conversely, high opportunity costs indicate high exhaustion (in activities categorized as work) or low recovery (in activities categorized as leisure). The continuous cost–benefit analysis during activity pursuit results in the decision to continue or abandon the current activity (Boksem & Tops, 2008; Cardini & Freund, 2019; Kurzban et al., 2013).

### Summary of the process model

We exemplified the new model of exhaustion and recovery using the sample case of work- and leisure-related activities. We did so because there seems to be a generally shared belief that work is exhausting and leisure is recovering. However, the model is conceptualized as a general model of exhaustion and recovery. It builds on the assumption that the experience of exhaustion or recovery largely hinges on the subjective expectations of how exhausting or recovering an activity is depending on the association of the life domain with exhaustion and recovery. To be clear: If a person—contrary to the commonly shared belief that work is exhausting and leisure recovering—expects their work to be recovering and leisure to be exhausting (e.g., a person who works as a dog walker and has the hobby to play chess competitively), we assume that they will be more likely to experience the same activity as more exhausting when framed as leisure and more relaxing when framed as work. In other words, we propose that the subjective expectation is strongly related a person’s general beliefs that activities in certain life domains are mainly exhausting or recovering. Given that activities do not come with a tag specifying to which life domain they belong, people use certain cues to categorize them. Accordingly, the first part of the model conceptualizes certain cues that people use to categorize an activity as belonging to a certain life domain (e.g., more to work or to leisure). The second part addresses the consequences of this categorization on exhaustion and recovery. Importantly, the categorization activates the belief that activities belonging to the respective life domain are mainly exhausting or recovering. These beliefs then selectively guide attention toward either signs of exhaustion or recovery. The relationships between several motivational factors (i.e., subjective value, energy expenditure, opportunity costs) account for the perceived degree of exhaustion (in activities categorized as work) or recovery (in activities categorized as leisure). These dynamic relationships are most likely unconscious and occur automatically (Boksem & Tops, 2008; Kurzban et al., 2013).

## The Competing Approach from Resource Accounts

A plausible alternative hypothesis is that exhaustion and recovery are not driven by subjective expectations but instead by activity characteristics (e.g., difficulty) and the amount of invested effort (or energy). In fact, resource accounts of exhaustion assume that engaging in an activity depletes (common or specific inner) resources that need to be replenished (through recovery). Depending on the expended amount of effort or energy, each activity consumes more or less of a limited resource that results in a state of resource depletion, experienced as exhaustion. Albeit criticized on theoretical and empirical grounds (e.g., Carter & McCullough, 2013; Hagger et al., 2010), resource models such as the ego-depletion model (e.g., Baumeister et al., 1994) still receive a lot of research attention (e.g., Inzlicht & Berkman, 2015), and the popular resource metaphor also guides current models of effort (e.g., Richter et al., 2016) and lay theories of exhaustion (e.g., Clarkson et al., 2010; Job et al., 2010).

In contrast to resource models, the present process model assumes that exhaustion and recovery are *motivational* signals (e.g., Inzlicht & Schmeichel, 2012; Kurzban et al., 2013) to disengage from an activity because it does not serve the attainment of the intended outcome to the degree that it justifies the current investment of goal-relevant means (Cardini & Freund, 2019). If the cost–benefit ratio of an activity turns unfavorable, people disengage from the given activity (to turn to a different activity with a more favorable cost–benefit ratio; for an excellent review on limited resource and motivational approaches, including their empirical evidence, see D. R. Evans et al., 2016).

Applied to the question of exhaustion and recovery in the work and leisure domain, resource accounts would predict that work-related activities consume more of a limited (inner) resource than leisure activities. Work activities deplete inner resources, whereas leisure activities restore them. By comparison, the present model of exhaustion and recovery posits that not activities per se but the categorization of a given activity as belonging to work or leisure impacts whether an activity is exhausting or recovering (following the widely shared expectations that work is exhausting and leisure is recovering).

One might argue that work-related activities are more exhausting because they are often pursued for longer periods of time and with more repetitions. However, this argument is again built on the assumption of a limited resource that gets depleted through repeated use over time. The longer one pursues an activity (e.g., chefs cooking for several hours), the more energy it consumes and the greater the resource depletion (Baumeister et al., 1994). However, people often experience exhaustion without prior resource depletion or no exhaustion after resource depletion (e.g., D. R. Evans et al., 2016). Moreover, people may pursue some of their—quite effortful—leisure activities such as jogging, cooking elaborate meals, or learning a musical instrument for hours and still feel recovered afterwards (e.g., Nakamura & Csikszentmihalyi, 2009). Cardini and Freund (2019) argued that subjective time perception (i.e., slower vs. faster momentary passage of time) rather than the objective duration of activity pursuit serves as an indicator of exhaustion. In addition, having low autonomy regarding the duration of an activity makes its categorization as work more likely, which, in turn, directs attention toward signs of exhaustion.

Ultimately, it is an empirical question whether exhaustion (and recovery) indicate the availability of resources or serve the motivational function to regulate the engagement in a given activity. In our view, the best route to empirically test the evidence for the effects of the hypothesized expectations (e.g., the same activity is more exhausting when categorized as work rather than leisure) against the competing limited resource models is to frame the very same activity as work once and as leisure at another and keep important activity characteristics (e.g., demands, duration) constant across work versus leisure.

## Future Directions

How can a person perceive the same activity to be recovering at one time and exhausting at another? We argue that the categorization of an activity as belonging to a certain life domain activates subjective expectations about how exhausting or recovering it will be and that these expectations then guide attention toward signs of exhaustion and recovery. The selective attention to signs of exhaustion increases the likelihood of experiencing exhaustion, whereas attending to signs of recovery probably intensifies feelings of recovery. Given the strong association of work with exhaustion and leisure with recovery, our model predicts that categorizing an activity as belonging to work will increase the likelihood of experiencing exhaustion, whereas an activity belonging to leisure will increase the likelihood of experiencing recovery. The model provides new and empirically testable hypotheses to better understand processes of exhaustion and recovery.

The aim of this article was to present a new process model of exhaustion and recovery, and we exemplified this model as it applies to the work and leisure context. Adopting a motivational view, the model, more generally, posits that categorizations of activities activate expectations that selectively guide attention to signs of either exhaustion or recovery. To this end, the model will stimulate innovative research in many psychological fields such as motivation, occupational psychology, and health psychology and inform practical applications to decrease exhaustion and increase recovery in people’s everyday lives (e.g., interventions taking advantage of expectations to increase beneficial effects). We discuss the theoretical and practical implications of the sample case of expectations about exhaustion and recovery in the work and leisure context first and of its extensions to other expectations about exhaustion and recovery afterward.

### Implications of the sample case of expectations in work and leisure

The process model, as outlined in this article, conceptualizes the sample case of expectations about exhaustion and recovery in the work and leisure context. The model conceptualizes the antecedents of the categorization of activities as belonging more to work and leisure and the consequences on exhaustion and recovery. In what follows, we outline the contributions of both parts to current research trends.

The model advocates the central role of the subjective categorization of activities to understand processes of exhaustion and recovery. The model does not attempt to search for types of activities (e.g., creative, manual, cognitive activities) or characteristics (e.g., difficulty, duration) that are more or less exhausting or recovering. Instead, the model is extremely parsimonious by positing that the subjective expectations related to the life domain to which an activity is perceived to belong are a main driver of experiences of exhaustion and recovery. However, we acknowledge that the question about the activity-specific variability merits further theoretical and empirical attention because the strength of the effects of the expectations might differ across activities and professional occupations.

Regarding the antecedents, the model proposes several cues that people use to categorize activities as belonging more to work or leisure. The cues are theoretically derived and primarily apply to the distinction between work and leisure but might also inform the distinction among other life domains (e.g., family). Notably, many psychological research fields build on the categorization of activities as belonging to one or more life domains. However, very little attention has been given to how people categorize activities as belonging to certain life domains (e.g., Inzlicht et al., 2014). The proposed cues provide the basis not only for investigating which cues people use to categorize activities into life domains but also for research addressing the interplay of life domains as conflicting or enriching each other (e.g., Amstad et al., 2011) and boundary drawing (e.g., notions about the separation and integration build on the conceptual distinction of life domains; Allen et al., 2014). The next step is to empirically test the relative importance of each cue for the distinction between work and leisure (e.g., Schüttengruber & Freund, 2022). For instance, if a person weighs the cue of autonomy higher for indicating leisure than the goal of the activity, they might feel that they pursue a leisure activity (with the associated expectation of recovery) when autonomy is high, whereas a person pursuing the very same activity but weighing more strongly the cue of the goal related to work might feel exhausted (in line with the associated expectation of work as exhausting). This might shed light on individual differences in the experiences of work- and leisure-related activities.

Adding to this discussion, dispositional factors of motivation and personality might not be best suited to understand how people categorize activities. Dispositions are, by definition, person characteristics, not characteristics of activities. Hence, a person who is generally motivated to avoid effort might also expect all activities to be more exhausting than a person who likes to expend effort. Thus, we would expect interindividual differences across activities, but it would not help us to identify intraindividual differences between activities. Similarly, people susceptible to social evaluation feel social evaluative threat not only in work but also in other life domains such as leisure (e.g., friends evaluating one’s music piece; sports trainers of team members evaluating one’s soccer performance). For others, social evaluation might not play a role in any life domain. Compared with the proposed cues, dispositional factors such as affiliation, achievement, and power motivation (McClelland, 1987) or personality traits such as extraversion or neuroticisms are relatively stable internal factors that influence people’s experiences regardless of the context. Future research should empirically support this by testing dispositional factors of motivation and personality against the cues proposed in the present process model.

The approach to focus on the categorization of activities to life domains that are associated with subjective expectations of exhaustion and recovery also opens new avenues for interventions preventing work-related exhaustion by highlighting cues of the activity that are associated with leisure (e.g., Crum & Langer, 2007). Similarly, when designing interventions for increasing health-related behaviors (e.g., physical exercise, adhering to a certain diet and preparing the related food), cues stressing the work-like nature of the activities (e.g., “your morning exercise is your first appointment of the day”) might lead to feeling more exhausted than when stressing the cues indicative of leisure (e.g., “allow yourself to take time off from your work schedule and start the day with your morning exercise”). Note, however, that people might be more likely to skip interventions categorized as leisure because work is often seen as more obligatory than the “nice-to-do” leisure activity. We encourage researchers to investigate the impact of participants’ subjective framing of study participation as work or leisure on long-term adherence, exhaustion, recovery, and other health indicators.

The framing of an activity as work or leisure is not completely arbitrary given the limits to the manipulation of the cues to change the categorization of activities as belonging more to work or leisure. To what degree one can “reframe” an activity as belonging to either work or leisure depends on the interindividual differences in the cue strength (e.g., switching locations might not change the categorization of processing reimbursements when the work goal outweighs the importance of the location) and one’s professional occupation (e.g., only chefs but not hairdressers could frame the activity “cutting vegetables” as work).

A research question in the area of occupational psychology—particularly with the increase of working from home since the COVID-19 pandemic—is to what degree the workplace and the home environment activate expectations that work is exhausting and leisure is recovering. Results might inform recommendations that allow to intentionally make use of people’s expectations. The manipulation of the cues (e.g., to create a more leisure-like office space) could help create workspaces that decrease exhaustion and increase recovery. Based on the ample evidence of the impact of expectations on psychological functioning (e.g., Job et al., 2015; Zion & Crum, 2018), studies on the effects of work and leisure framings are a promising route to not only better understand processes of exhaustion and recovery but also create innovative interventions in the service of a successful work–nonwork balance. We hasten to add the caveat that we do not endorse simply reframing work as leisure and thereby eliminating that people feel exhausted when working a lot. There seems to be a trend to create workplaces that resemble leisure contexts by including playful and home-like environments, thereby suggesting to employees that they are “not really” working. Although it might help against feeling exhausted by work, it seems like a slippery slope to blur the lines of work and leisure to a degree that it becomes difficult for employees to insist on a clear set of work hours. Thus, creating interventions that prevent exhaustion from work requires great care not to simply reframe activities as “leisure” (instead of “work”).

### General implications of expectations about exhaustion and recovery

Moving beyond the sample case of work and leisure, there are many widely shared, yet understudied expectations that revolve around the categorization of activities and influence people’s sensitivity toward signs of exhaustion or recovery. For example, people might associate low (compared with high) perceived autonomy with less exhaustion and more recovery (e.g., “If I have to engage in this activity, it will certainly be exhausting”; Csikszentmihalyi & LeFevre, 1989; Inzlicht et al., 2014). In this example, the categorization of autonomy as being low (compared with high) selectively guides people’s attention toward signs of exhaustion (instead of recovery). The process model sheds light on the many—yet to be explicated—expectations that people hold about exhaustion and recovery. Future research can transfer the presented process model to theoretically discuss and empirically study these expectations.

Although expectations about exhaustion and recovery might be most pronounced for activities in the domains of work and leisure, people likely also hold expectations about exhaustion and recovery in other life domains, albeit characterized by higher interindividual variability. For example, some people might expect activities categorized as belonging to the domain of the household to be exhausting (e.g., ironing), whereas others might expect these activities to be recovering (e.g., gardening). Family-related and social activities might be expected to be recovering when experienced as leisure (e.g., family trip to cinema) and to be exhausting when experienced as work (e.g., childcare duties). Expectations related to other life domains can be reduced to the experience of the given activity as more work-related versus leisure-related, in line with the parsimony of the presented model.

Expectations might change across the life span. When juggling the many demands of work and parenthood in middle adulthood, people might expect family-related activities to be exhausting, whereas they might expect the same activities to be recovering in older adulthood (e.g., grandparenthood; Freund, 2020). Even individual associations of life domains with exhaustion or recovery follow the same logic of selectively guiding people’s attention toward signs of either exhaustion or recovery.

Turning to the cues for the categorization of activities, the cues also apply to categorizing activities as belonging to life domains other than work and leisure. For example, the prototypical family activity is not paid, serves a family goal, is pursued outside “usual” (depending on the profession) work hours and outside the “usual” (depending on the profession) workplace, together with family. Autonomy is high or low, depending on whether the activity is perceived as obligatory (similar to work). These transfers to other life domains are subjects for future research.

Occupational psychology might profit from considering the impact of potential expectations on exhaustion and recovery to better understand and increase the benefits of psychological detachment as the core recovery process (e.g., Sonnentag, 2012). For instance: Is detachment from work easier when engaging in activities with particularly strong cues for leisure because people expect them to be recovering? Many people seem to be able to detach from work with work-like activities (e.g., carpentering, gardening), and these activities might detach them primarily because they are categorized as leisure.

Given these practical implications, an important caveat is that the model does not address chronic states of exhaustion as a core dimension of job burnout or the accumulation of repeated temporal to chronic exhaustion related to long-term impairments of physical and mental health, as discussed in the occupational and clinical literature (e.g., Demerouti et al., 2001, 2021; Maslach et al., 2001; Meijman & Mulder, 1998; Sonnentag et al., 2017). Rather, the model focuses on exhaustion and recovery while engaging in a given activity categorized as either work or leisure. Potential applications to longer time periods and accumulated exhaustion and recovery are intriguing research questions. For example, people might hold expectations about how much time one should spend on activities categorized as work compared with belonging to other life domains to achieve a good “work-life balance” (e.g., time equally dedicated to activities categorized as work, leisure, family). If people do not meet their expectation of the “optimal” time allocation for a good work–life balance, they might expect—and experience—signs of chronic exhaustion and disengagement from work-related activities, which are both dimensions of burnout (e.g., Demerouti et al., 2001), instead of regular phases of recovery (e.g., relaxing evening or weekends).

Another important venue for future research is the investigation of the role of expectations underlying the preference to separate or integrate life domains. Expectations about exhaustion and recovery might yield insights into the relationship between boundary drawing and outcomes of exhaustion and recovery (Allen et al., 2014). For instance, if talking with friends about work is considered a work-related activity, people might feel exhausted and prefer to keep discussions about work out of their friendship activities. In contrast, if discussing a work project with colleagues after work in a bar for the purpose of building a friendship with them, it might be experienced as recovering. In this case, an integration of life domains might be experienced as enriching rather than exhausting.

### Conclusion and outlook

Adopting a motivational view of exhaustion and recovery, the proposed model posits dynamic relationships between several motivational factors that account for the degree of exhaustion or recovery. Toward that end, the model draws on several motivational approaches such as opportunity costs (Cardini & Freund, 2019; Kurzban, 2016), subjective value (Berkman et al., 2017), motivational intensity theory (e.g., Gendolla et al., 2019; Richter et al., 2016), confirmation bias (J. S. B. T. Evans, 2016; Nickerson, 1998), and research on mindsets (e.g., Zion & Crum, 2018) to move beyond the mere description of the effects of expectations on exhaustion and recovery. The process model extends existing motivational approaches and highlights their importance for understanding expectations about exhaustion and recovery. We argue that neither person characteristics nor the demandingness of the activity are the main factors determining how exhausting or recovering people perceive it. The challenge is to empirically disentangle the effects of subjective expectations (i.e., effect of the categorization of activities as work or leisure) from differences in activity pursuits (e.g., high vs. low demands). Separating the effects will advance research on processes of exhaustion and recovery.

In this article we mainly focused on the sample case of work and leisure. However, the model is open to a wide range of transfers to other expectations about exhaustion and recovery in psychological research areas such as motivation, occupational, and health psychology. The guiding question is to what degree do categorizations of activities activate different expectations of exhaustion and recovery that, in turn, influence people’s sensitivity towards signs of exhaustion or recovery. Our process model attempts to provide a theoretical and empirically testable framework to answer this question. We hope that other researchers will build on, sharpen, and modify the process model in the shared goal of better understanding processes of exhaustion and recovery.

## Transparency

*Action Editor:* Tina M. Lowrey

*Editor:* Klaus Fiedler

## Notes

1. The terms “fatigue” and “exhaustion” are often used interchangeably (Frone & Tidwell, 2015). We focus on cognitive exhaustion because most people’s work consists of nonphysical activities that vary in how cognitively demanding they are.

2. The terms “expectation,” “mindset,” and “belief” are used interchangeably in much of the literature (see Zion & Crum, 2018).

3. “Energy” and “effort,” although distinct constructs with “energy” in psychology often used analogously to physics, and “effort” denoting the intensity with which people pursue an activity, are often used interchangeably (e.g., Richter, 2013; Richter et al., 2016).
